# Supra-Pubic Lithotomy, a Case

**Published:** 1891-08

**Authors:** Will B. Davis

**Affiliations:** City Physician, Puebla, Col.


For Daniel's Texas Medical Journal.

### SUPRA—PUBIC LITHOTOMY.

BY WILL B. DAVIS, M. D., CITY PHYSICIAN, PUEBLA, COL.

[Abstract of report of a case, operated on Feb. 25th, 1891.]

JACK McCLEES, aged 28, locomotive engineer, a native of Scotland, had suffered more or less, since about 15 years of age, with bladder trouble; but for about a year previous to operation had been almost totally disabled. Stone in the bladder was readily diagnosed. On the 25th of February the supra pubic section was done, at 11 a. m.; the stone, an oxalate of lime calculus, measured 5¾ inches in its greatest and 4⅞ inches in its smallest diameter. It was one of the largest and prettiest specimens of the "mulberry" I ever saw.

An hour after operation the pulse was 140, temperature 97¾°; at 7:00 p. m. pulse had fallen to 100, and the temperature had risen to 100°. Pulse and temperature fluctuated from normal—the former to 120, and the latter to 100¾° during the three or four days following, showing at no time any active disturbance. The maximum temperature at any time did not exceed 100⅓°. [Daily record of pulse and temperature omitted.—ED.]

No stitches were taken in the bladder; but the upper half of the wound was closed with deep and superficial sutures; and before withdrawing my index finger from the bladder, two drainage tubes had been placed, one a No. 36 F. velvet catheter, the other an ordinary drainage tube, small size. These were secured in lower angle of incision by stitch. The tubes after passing through the dressings of iodoform gauze and absorbent cotton, were passed over the pubis and into a bed-pan between the thighs. The drainage was so perfect, that the dressings never became soiled, either from urine or from the borax wash which was used at every visit; by means of a Van Buren bag it was an easy matter to thoroughly cleanse the bladder, passing through it as much water as was necessary. The patient could change position at will; and on the sixth day the tubes were withdrawn and patient dismissed. On the 10th day after operation, he was able to go to the dining room to his meals without assistance. By the 15th the incision had entirely closed. Of course, the catheter had to be used frequently, and the patient readily learned to introduce it. On the 28th day after operation patient reported for duty at the railroad office.

During confinement, it is unnecessary to say, the bowels were attended to, as were all other matters of personal hygiene. The dressings were removed and changed several times; more from custom than from necessity, and to enable me to observe the "behavior" of the wound.

I am partial to the supra-pubic operation, since I am thoroughly convinced that there is no necessity of invading the peritoneum; nor is there as much danger, if any, of infiltration of the urine, provided thorough drainage is secured. Besides, when the patient recovers, he is *well*; which cannot be said if the perineal operation has been performed; for many cases which I have seen (of perineal operation) reported as "recovered" (i. e. they didn't *die*), were troubled with incontinence of urine, seminal drain, and not a few with permanent fistula; though in the latter cases I am persuaded the patients were the subject of tubercle of the prostate.

With warmest regards to my many friends in the Texas State Medical Association, I hope they will accept this, from the foot of Pike's Peak, in the same kind and indulgent spirit they have ever shown my efforts.

[It will be remembered that while Dr. Davis was residing in Tarrant county, Texas, he contributed a paper to the State Medical Association, on the treatment of hemorrhoids by injections of carbolic acid. The paper was read at Tyler, in 1882, and is published in the transactions of that year. The paper was the feature of the meeting, and created a profound sensation, it was something new then; and Dr. Davis' success was unprecedented. Dr. John A. Wythe was present, and criticised the paper, saying that if all surgeons could secure the results reported by Dr. D., the problem of hemorrhoids was forever solved, or words to that effect. We hope to hear from the doctor again.—Ed.]

